# Estimation of the genetic parameters for semen traits in Chinese Holstein bulls

**DOI:** 10.1186/s12863-019-0752-x

**Published:** 2019-06-10

**Authors:** Hongwei Yin, Lingzhao Fang, Chunhua Qin, Shengli Zhang

**Affiliations:** College of Animal Science and Technology, Key Laboratory of Animal Genetics and Breeding of Ministry of Agriculture, National Engineering Laboratory of Animal Breeding, China Agricultural University, Beijing, 100193 China

**Keywords:** Semen traits, Genetic parameters, Chinese Holstein bulls

## Abstract

**Background:**

Semen traits are important for the widespread use of superior bulls. Thus, the objective of this study was to estimate the heritability of five semen traits, ejaculate volume (VE), progressive sperm motility (SM), sperm concentration (SC), number of sperm (NSP), and number of progressive motile sperm (NMSP), and their genetic correlations (*r*_*g*_). The dataset being studied consisted of 1450 Chinese Holstein bulls with full pedigree information, born between 1996 and 2012, representing 11 AI centers. Genetic parameters were estimated in a multivariate analysis using the average information restricted maximum likelihood estimation of variance (AI-REML).

**Results:**

The estimates of heritability for VE, SM, SC, NSP, and NMSP were 0.15, 0.12, 0.22, 0.16 and 0.12, respectively. The genetic correlations among the five semen traits ranged from 0.02 (VE and SC) to 0.99 (NSP and NMSP).

**Conclusions:**

Our findings provide useful information on the heritability of semen traits in Holstein bulls and the relationships among them, and should assist in selection for improvement of semen traits in Chinese Holstein bulls.

## Background

Female fertility has been widely studied for breeding purposes in many countries [1–3], whereas less focus has been placed on male fertility. In cattle, male fertility can be measured directly on semen or indirectly from females. Several studies have been conducted on bull fertility [4–8]. Unlike the male fertility traits measured from females, semen traits are measured directly in males and can be used as indicators of male fertility. Semen traits are complex and are influenced by genetic and environmental factors, such as season, age, bull handlers, and the interval between consecutive semen collections in days [9–11]. The first published estimates of the heritability of semen traits were low to medium (ranging from 0.02 to 0.31) [12], and the heritability of semen traits has been studied in many cattle populations [7, 10, 13–16]. However, there were big differences in the estimates from these studies.

Conception rate in the Holstein cattle has declined dramatically recently in some countries [3, 17]. Because conception rate is affected by semen characteristics such as sperm volume and sperm motility, the estimation of genetic parameters for semen traits can offer important information that could be used to improve the conception rate in Holstein dairy cattle. However, since the first AI center was built in China (1973), there has been little focus on semen traits, and no studies have been done on semen quality or the genetic parameters of semen traits in Chinese Holstein bulls. Therefore, the objectives of this study were: 1) to estimate the heritability of semen traits in Chinese Holstein cattle, and 2) to estimate the genetic and phenotypic correlations among semen traits. The results will help us better understand the genetic basis of semen traits, contributing to their genetic improvement in Holstein cattle.

## Materials

**Fig. 1:**
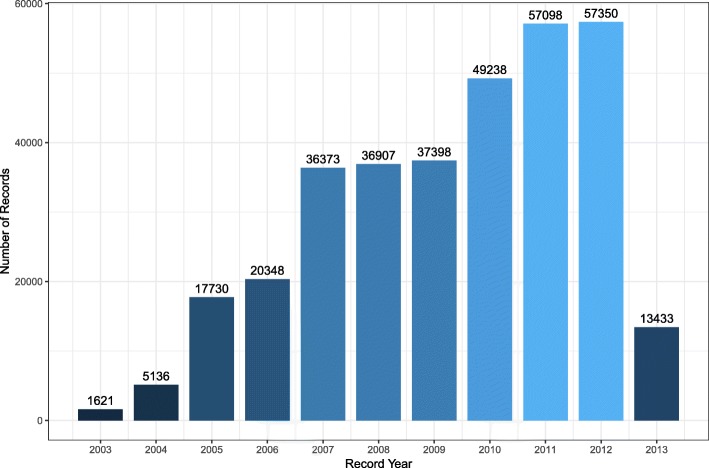
The distribution of data across record years

Data from a total of 1450 Chinese Holstein bulls were used in this study. The bulls were from 11 artificial insemination (AI) centers in China, and the number of bulls in each center ranged from 69 to 202. All the bulls were born between 1996 and 2012, and five semen traits were recorded across years (Fig. 1), ejaculate volume (VE) (mL) was read directly from a graduated collection tube (graduation: 1/10 mL); progressive sperm motility (SM) was the proportion of forward moving sperm, which was evaluated under microscope by experienced technicians; sperm concentration (SC) was measured using a spectrophotometer; number of sperm per ejaculate (NSP) was calculated by multiplying VE and SC, and the number of motile sperm (NMSP) was calculated by multiplying NSP and SM. In each AI center, ejaculations were conducted once or twice per day, often by different semen handlers, and the interval between the consecutive collections for each bull was 30 min to 1 h. The approaches used for semen collection were standardized across AI centers.

The pedigree information was extracted from the Dairy Data Centre of China (http://www.holstein.org.cn/) by tracing back as many generations as possible for the bulls. The number of animals in the pedigree file was 12,985.

## Methods

For each of the five semen traits, the model for the genetic parameters was:

$$ {\mathrm{y}}_{\mathrm{g}\mathrm{hijklmno}}=\upmu +{\mathrm{RecYea}}_{\mathrm{g}}+{\mathrm{BulAge}}_{\mathrm{h}}+{\mathrm{Sea}}_{\mathrm{i}}+{\mathrm{ColInt}}_{\mathrm{j}}+{\mathrm{Num}}_{\mathrm{k}}+{\mathrm{BulSta}}_{\mathrm{l}}+{\mathrm{BulHan}}_{\mathrm{m}}\ \left({\mathrm{BulSta}}_1\right)+{\mathrm{a}}_{\mathrm{n}}+{\mathrm{p}}_{\mathrm{o}}+{\mathrm{e}}_{\mathrm{g}\mathrm{hjklmno}} $$

where *y*_*ghijklmno*_ is the record of the semen phenotype; *μ* is the overall intercept; *Recyea*_*g*_ is the fixed effect of the record year from 2003 to 2013; *BulAge*_*h*_ is the fixed effect of age of bull at collection, where the five age classes (h = 1 to 5) were defined as 10–24 months, 25–36 months, 37–60 months, 61–96 months, and older than 96 months; *Sea*_*i*_ is the fixed effect of the season of collection, where the four seasons (i = 1 to 4) were defined as December to February, March to May, June to August, and September to November; *ColInt*_*j*_ is the fixed effect of the interval of between consecutive collections for each bull (j = 1 to 5), which was defined as 0 d, 1–4 d, 5–7 d, 7–11 d, and longer than 11 d; *Num*_*k*_ is the fixed effect of the number of collections on the respective collection day (k = 1 to 2); *BulSta*_*l*_ is the fixed effect of AI station (l = 1 to 11); *p*_*o*_ is the random permanent environment effect with var.(*p*) ~ *N(*0, *Iσ*^*2*^_*pe*_), where *σ*^*2*^_*pe*_ is the permanent environmental variance and *I* is the identity matrix; *a*_*n*_ is the random additive genetic effect with var.(*a*) ~ *N(*0, *Aσ*^*2*^_*a*_), where *σ*^*2*^_*a*_ is the additive genetic variance and A is the relationship matrix; *BulHan*_*m*_ (*BulSta*_*l*_) is the effect of *BulHan*_*m*_ (m = 1 to 54), as a random effect of bull handlers, nested within AI station; *e*_*ghijklmno*_ is the random residual effect with var.(*e*) ~ *N(*0, *Iσ*^*2*^_*e*_), where *σ*^*2*^_*e*_ is the residual variance. (Co) variance components were estimated using the DMU software package [20] with the average information restricted maximum likelihood (AI-REML) [21].

## Results and discussions

The total number of ejaculates was 332,632 and the average number of recorded ejaculates per bull was 229. The bulls had an average semen volume of 7.22 mL, progressive sperm motility of 67%, sperm concentration of 11.37 × 10 9/mL, total sperm of 83.52 × 10 9, and total motility sperm of 59.21 × 10 9 (Table 1). There was a significant difference in the five semen traits among the 11 AI centers (*P* < 0.01).

**Table 1:** Means (SD) for each ejaculate (ejaculate volume (VE), progressive sperm motility (SM), sperm concentration (SC), number of sperm (NSP), and number of progressive motile sperm (NMSP) among 11 AI centers using GLM in SAS

AI station	VE	SM	SC	NSP	NMSP
1	9.68 ± 4.32	0.72 ± 0.09	11.30 ± 3.93	107.10 ± 52.91	77.72 ± 40.29
2	5.63 ± 2.44	0.61 ± 0.15	8.68 ± 5.19	50.08 ± 40.99	31.13 ± 27.01
3	5.52 ± 2.16	0.43 ± 0.15	10.41 ± 3.56	58.11 ± 30.94	25.83 ± 16.80
4	8.34 ± 2.76	0.82 ± 0.06	13.81 ± 4.62	112.92 ± 47.17	92.81 ± 39.23
5	4.68 ± 2.55	0.69 ± 0.16	9.01 ± 4.61	42.73 ± 33.32	30.77 ± 24.21
6	7.89 ± 2.16	0.65 ± 0.05	13.07 ± 4.01	103.76 ± 42.98	68.00 ± 29.43
7	6.94 g ± 2.27	0.68 ± 0.10	11.64 ± 4.01	80.57 ± 37.54	55.24 ± 27.22
8	6.89 g ± 2.33	0.55 ± 0.18	11.49 ± 3.62	78.68 ± 34.64	45.26 ± 26.71
9	5.71 ± 2.10	0.63 ± 0.07	9.23 ± 3.50	52.44 ± 27.57	33.33 ± 17.87
10	5.71 ± 2.24	0.57 ± 0.17	10.26 ± 5.31	58.56 ± 37.97	35.53 ± 24.96
11	8.17 ± 2.95	0.85 ± 0.06	15.11i ± 4.53	124.80 ± 60.52	107.04 ± 52.23
Average	7.22 ± 3.41	0.67 ± 0.17	11.37 ± 4.74	83.52 ± 52.31	59.21 ± 43.45

The estimated genetic parameters for semen traits are presented in Table 2. The heritability estimates for VE, SM, SC, NSP, and NMSP were 0.15, 0.12, 0.22, 0.16, and 0.12, respectively. All the estimates were significantly greater than zero as shown by the standard error of these estimates (Table 2). A low genetic correlation (0.02) was found between VE and SC. SM had a moderate genetic correlation with all the other traits (ranged from 0.18 to 0.36). The genetic correlations among all other semen traits were high (from 0.65 to 0.99). Similarly, a low phenotypic correlation (0.09) was also found between VE and SC. SM had a moderate phenotypic correlation with all the other traits (ranged from 0.26 to 0.56). The phenotypic correlations among the other semen traits were high (from 0.63 to 0.96). All phenotypic correlations were in the same direction as the corresponding genetic correlations.

**Table 2:** Heritability (on diagonal, SE in parentheses), genetic correlations (below diagonal, SE in parentheses) and phenotypic correlations (above diagonal) for each ejaculate (ejaculate volume (VE), progressive sperm motility (SM), sperm concentration (SC), number of sperm (NSP), and number of progressive motile sperm (NMSP)

traits	VE	SM	SC	NSP	NMSP
VE	0.15 (0.03)	0.26	0.09	0.75	0.71
SM	0.36 (0.21)	0.12 (0.03)	0.35	0.39	0.56
SC	0.02 (0.12)	0.18 (0.16)	0.22 (0.04)	0.66	0.63
NSP	0.70 (0.25)	0.25 (0.17)	0.71 (0.26)	0.16 (0.03)	0.96
NMSP	0.74 (0.28)	0.25 (0.19)	0.65 (0.26)	0.99 (0.39)	0.12 (0.03)

In general, our heritability estimates for VE, SC, SM, NSP, and NMSP ranged from 0.12 to 0.22, which implies that there was a substantial environmental influence on these traits [14]. Our heritability estimate for VE was consistent with previous studies [7, 10, 14, 18]. However, our value was lower than the estimate reported by Ducrocq et al. (1995) (0.65), both those estimates were based on mean values from more than 10 records per individual in young Normande bulls. The heritability for SM (0.12) was also in accordance with the results of some previous studies [12, 14, 19], but was lower than estimates from others [7, 10, 15, 16]. These differences may be due to different breeds, genetic backgrounds, and different models used for parameter estimation. Fewer heritability estimates have been published for NSP and NMSP. For NSP, heritability was estimated to be 0.09 by Druet et al. (2009), 0.22 by Gredler et al. (2007), and 0.24 by Knights et al. (1984), which are close to our estimate of 0.16. For NSP and NMSP, our heritability estimates are 0.16 and 0.12, respectively,which are much lower than the 0.38 and 0.49 for young bulls, and 0.54 and 0.64 for older bulls reported by Mathevon et al. (1998), which was based on a small sample size of198 bulls separated into young bulls (up to 30 months old) and older bulls (between 4 and 6 years old) [10]. The heritability of NMSP (0.12) was higher than the 0.04 value reported by Gredler et al. (2007). This difference may be attributed to a different breed—their breed was Austrian dual-purpose Simmental (Fleckvieh)—and the sample size in their study (12,746 ejaculates from 301 bulls) was smaller than in this study [15]. The heritability of 0.22 for SC was in accordance with values from other studies on Holstein bulls [7, 18].

In general, the genetic correlations among all five semen traits were positive. The genetic correlation between VE and SM (0.36) was similar to the value of 0.31 reported by Gredler et al. (2007), whereas the negative correlations of − 0.20 and − 0.38 were reported by Druet et al. (2009) and Kealey et al. (2006), respectively. The genetic correlation between SC and NSP (0.46, s.e. 0.18) reported by Druet et al. [7] was lower than the estimate of 0.71 (s.e. 0.16) found in the present study; the estimates for the genetic correlation between SC and SM differed only slightly between these studies (0.12 v 0.18, respectively) [7]. The results of genetic correlations in our study suggest that selection for one semen trait should have no unfavourable effects on the other semen traits measured.

The low heritability of semen traits (0.12 to 0.22) indicates a substantial environmental influence; this suggests that potential environmental factors that may affect semen quality, such as bull handler, season, and the interval between semen collections need to be controlled carefully so that genetic differences are more accurately assessed. Therefore, to improve semen quality and increase the quantity of superior bulls, environmental influences such as bull handlers, season, and the interval of two continual semen collections for each bull, should be optimized. In addition, other environmental influences that are not routinely recorded in AI stations, such as temperature, humidity, and feed, should be recorded, and studies of candidate genes for semen traits and GWAS can be performed with the aim of selecting bulls with superior semen quality and quantity.

## Conclusions

In this study, we estimated the genetic parameters of five semen traits in Chinese Holstein bulls for the first time. The heritability of semen traits was varied from 0.12 to 0.22. The correlations among the semen traits were all positive. NSP had a strong genetic correlation with all other semen traits and its heritability was 0.16, which suggests the focus of genetic improvement ought to be around NSP. Our results provide valuable insights into the genetic basis of semen traits in the production period and should assist in designing selection programs to improve semen traits in Chinese Holstein bulls.

## Data Availability

The datasets analyzed during the current study are available from the corresponding author on a reasonable request.

## Abbreviations

NMSP: The number of motile sperm () was calculated by multiplying NSP and SM
NSP: Number of sperm per ejaculate was calculated by multiplying VE and SC
SC: Sperm concentration was measured using a spectrophotometer
SM: Progressive sperm motility was the proportion of forward moving sperm, which was evaluated under microscope by experienced technicians
VE: Ejaculate volume (mL) was read directly from a graduated collection tube (graduation: 1/10 mL)
